# CDK4 is an essential insulin effector in adipocytes

**DOI:** 10.1172/JCI170315

**Published:** 2023-03-15

**Authors:** Sylviane Lagarrigue, Isabel C. Lopez-Mejia, Pierre-Damien Denechaud, Xavier Escoté, Judit Castillo-Armengol, Veronica Jimenez, Carine Chavey, Albert Giralt, Qiuwen Lai, Lianjun Zhang, Laia Martinez-Carreres, Brigitte Delacuisine, Jean-Sébastien Annicotte, Emilie Blanchet, Sébastien Huré, Anna Abella, Francisco J. Tinahones, Joan Vendrell, Pierre Dubus, Fatima Bosch, C. Ronald Kahn, Lluis Fajas

Original citation: *J Clin Invest*. 2016;126(1):335–348. https://doi.org/10.1172/JCI81480

Citation for this retraction: *J Clin Invest*. 2023;133(6):170315. https://doi.org/10.1172/JCI170315

At the request of the corresponding author, the *JCI* is retracting this article. The authors became aware that the genotype of the mice described as *Cdk4^neo/neo^ Rip-Cre* was incorrect. The correct mouse genotype was *Cdk4^neo/neo^ Rip-Cdk4^R24C^*. The model used transgenically expresses the *Cdk4^R24C^* allele under the control of the rat insulin promoter (*Rip-Cdk4^R24C^*) rather than expressing *Cdk4^R24C^* in the *Cdk4* locus as described in the article. Due to this unintentional error, the corresponding author requested retraction.

## Version 1 03/15/2023

Electronic publication

